# Functionalization of silk with actinomycins from *Streptomyces anulatu*s BV365 for biomedical applications

**DOI:** 10.3389/fbioe.2024.1466757

**Published:** 2024-09-19

**Authors:** Tatjana Ilic-Tomic, Ana Kramar, Mirjana Kostic, Sandra Vojnovic, Jelena Milovanovic, Milos Petkovic, Paul M. D’Agostino, Tobias A. M. Gulder, Jasmina Nikodinovic-Runic

**Affiliations:** ^1^ Institute of Molecular Genetics and Genetic Engineering, University of Belgrade, Belgrade, Serbia; ^2^ Department of Textile Engineering, Faculty of Technology and Metallurgy, University of Belgrade, Belgrade, Serbia; ^3^ Novel Materials and Nanotechnology Group, Institute of Agrochemistry and Food Technology (IATA), Spanish Council for Scientific Research (CSIC), Paterna, Spain; ^4^ Faculty of Pharmacy, University of Belgrade, Belgrade, Serbia; ^5^ Helmholtz Institute for Pharmaceutical Research Saarland (HIPS), Saarbrücken, Saarland, Germany; ^6^ Technical University of Dresden, Dresden, Saxony, Germany

**Keywords:** silk, antimicrobial, *Streptomyces*, actinomycins, nonactin, functional biomaterials, biocompatibility, anticancer

## Abstract

Silk, traditionally acclaimed as the “queen of fiber,” has been widely used thanks to its brilliant performance such as gentleness, smoothness and comfortableness. Owing to its mechanical characteristics and biocompatibility silk has a definitive role in biomedical applications, both as fibroin and fabric. In this work, the simultaneous dyeing and functionalization of silk fabric with pigments from *Streptomyces anulatus* BV365 were investigated. This strain produced high amounts of orange extracellular pigments on mannitol-soy flour agar, identified as actinomycin D, C2 and C3. The application of purified actinomycins in the dyeing of multifiber fabric was assessed. Actinomycins exhibited a high affinity towards protein fibers (silk and wool), but washing durability was maintained only with silk. Acidic condition (pH5) and high temperature (65°C) facilitated the silk dyeing. The morphologies and chemical components of the treated silk fabrics were analyzed using scanning electron microscopy and Fourier transform infrared spectroscopy. The results showed the pigments bind to the silk through interaction with the carbonyl group in silk fibroin rendering the functionalized, yet surface that does not cause skin irritation. The treated silk exhibited a remarkable antibacterial effect, while the biocompatibility test performed with 3D-reconstructed human epidermis model indicated safe biological properties, paving the way for future application of this material in medicine.

## 1 Introduction

For decades, silk fibroin (SF) extracted from *Bombyx mori* silkworm cocoons has found applications in textiles, but also as suture material and surgical meshes in medicine (Zhang et al., 2024). Biomaterials and fabrics based on silk fibroin exhibit unique capabilities in promoting skin wound healing due to their hemostatic properties, low inflammatory potential, and penetrability to oxygen and water, as well as their capability to act as a barrier to bacterial colonization (Chirila, 2022; Ghalei and Handa, 2022; Holland et al., 2019; Janani et al., 2023). Dressings based on SF are offered as sponges, hydrogels, nanofibrous matrices and electrospun mats, scaffolds, micro/nanoparticles, and films, and are currently exploited for treating a chronic and acute wounds (Chouhan and Mandal, 2020; Mazurek et al., 2022; Patil et al., 2020; Vidya and Rajagopal, 2021). Furthermore, the SF in various types (such as hydrogel, micro/nanoparticles, and thin film) can be used to capture or adsorb a variety of bioactive molecules or nanoparticles (Zhu et al., 2024; Cao et al., 2023). For example, basic fibroblast growth factor (bFGF) loaded liposomes with silk fibroin hydrogel core prevent degradation of bFGF in wound fluid and accelerate the wound closure through the promotion of granulation tissue formation, collagen deposition, angiogenesis, and reepithelialization (Hu et al., 2017). Similarly, SF loaded with silver and antimicrobial peptide nanoparticles were described for antibacterial capability and enhanced osseointegration (Zhou et al., 2023a).

*Streptomyces* are aerobic Gram-positive bacteria with complex lifecycles that usually live in the soil, but inhabit a wide range of other ecological niches. *Streptomycetes* produce a plethora of so-called secondary metabolites with important functions. Compounds produced by *Streptomyces* include many antibiotics (e.g., streptomycin, gentamycin, actinomycin), anticancer compounds (e.g., doxorubicin, rapamycin) and other bioactive molecules (Alam et al., 2022). *Streptomyces* also create many pigment molecules such as roseophilins (Kawasaki et al., 2008) and prodigiosins (Kramar et al., 2014; Li et al., 2022; Sebastian et al., 2022; Stankovic et al., 2012), covering the whole spectrum from violet to yellow and red (Janković et al., 2023; Sarmiento-Tovar et al., 2022). The application of natural colors for textile dyeing is experiencing renaissance owing to the increase in ecological awareness and need to reduce the use of synthetic dyes, especially in the textile industry (Bechtold et al., 2003; Haddar et al., 2018; Ren et al., 2021).

A number of pigments from *Streptomyces* were shown to have antimicrobial and anticancer properties (Metwally et al., 2021; Rather et al., 2022) with potential applications as functional dyes. Actinomycins are a family of chromopeptides with strong cytotoxic and antibiotic activities produced by *Streptomyces* (Wang et al., 2017). They involve of an actinoyl chromophore (2-amino-4,6-dimethylphenoxazine-3-one-1,9- dicarboxylic acid) with two cyclic pentapeptide lactones linked to its carboxyl groups. Actinomycin D is recognized member of this family and has found clinical usage as an anti-cancer medicine for the treatment of juvenile rhabdomyosarcoma and Wilms’ tumor (Marchal et al., 1997).

Fabrics can harness molecular properties to incorporate integral functionalities, catering to specific end-use requirements beyond their primary purpose of dyeing. Functional dyes can be employed to achieve dual outcomes, providing both coloration and functional finishing effects, such as UV protection, antimicrobial properties, or antioxidant activity (Sun et al., 2013). Functional dyes of natural origin, that effectively interact with material surfaces and are sustainably produced are highly sought after.

The present study reports the isolation of the strain *Streptomyces anulatus* BV365 associated with the ectomycorhizosphere of the black truffle that showed remarkable yellow pigmentation. Several metabolites were isolated and identified from the fermentation broth, namely, actinomycins and nonactin, which exhibit antibacterial activity against pathogenic microorganisms in the nanograms range and strong cytotoxicity against human cell lines. Actinomycins were used for silk dyeing and the antibacterial and cytotoxic properties of dyed silk fabrics were evaluated. In addition, a skin irritation test using skin model was performed in order to assess the biocompatibility of the tested fabrics for future applications in medicine and healthcare applications.

## 2 Materials and methods

### 2.1 Chemicals and reagents

All commercial chemicals were of reagent-grade quality or higher and used without further purification. Solvents, including n-hexane, ethyl acetate, methanol, acetone were used as received (Sigma Aldrich, Steinheim, Germany).

Mannitol, tryptone, yeast extract, malt extract, tryptic soy broth and agar were purchased from Biolife, Milan, Italy, soy flour was purchased from local health food store. D-Glucose, ammonium chloride (NH_4_Cl), ferric(III) ammonium citrate [Fe(III)NH_4_-citrate], disodium phosphate (Na_2_HPO_4_x12H_2_O) and monopotassium phosphate (KH_2_PO_4_) were purchased from Fisher Scientific, Loughborough, United Kingdom. Sodium chloride (NaCl), calcium chloride (CaCl_2_x2H_2_O), dimethyl sulfoxide (DMSO), N-Z Amine, and (3-(4,5-dimethylthiazol-2-yl)-2,5-diphenyltetrazolium bromide (MTT) were purchased from Sigma Aldrich, Steinheim, Germany. Magnesium sulfate (MgSO_4_x7H_2_O) and ethyl acetate were purchased from CentroChem, Lublin, Poland.

Multifiber fabric style 49 (James Heal, England, United Kingdom) containing cellulose acetate, cotton, polyamide 6.6 (PA), polyester (PES), acrylic (PAN), silk, viscose and wool, was used. Individual fabrics used for experiments were provided by commercial suppliers and used as received; bleached greige silk (79.8 g m^−2^) provided by Bon Ami International Ltd. Calcutta, India.

Commercial washing detergent ECE Formulation Non-Phosphate Reference Detergent A- Without Optical Brightener Stock Codes 706-652 (EN ISO 105 C08 and C09) was supplied by SDC Enterprises Limited.

### 2.2 Isolation of a bacterial strain responsible for pigment production

*S. anulatus* BV365 was isolated from the ectomycorrhizosphere soil of the black truffle *Tuber melanosporum*, according to a previously described procedure using casein starch agar (Djokic et al., 2011). The strain was described by its morphological characteristics (mycelia, cell morphology, and spore surface) by observing the cultures on MSF plates (Mannitol 20 g L^−1^, Soy flour 20 g L^−1^, Agar 20 g L^−1^), ISP2 (International *Streptomyces* Project Agar; glucose 4 g L^−1^, yeast extract 4 g L^−1^, malt extract 10 g L^−1^, agar 20 g L^−1^, MSM (Minimal Salt Medium; Na_2_HPO_4_ × 12 H_2_O 9 g L^−1^, KH_2_PO_4_ 1.5 g L, NH_4_Cl 1 g L^−1^, MgSO_4_ × 7H_2_O 0.2 g L^−1^, CaCl_2_ × 2H_2_O 0.2 g L^−1^, trace elements solution 0.1%, N–Z amine 0.025%, agar 20 g L^−1^ and glucose 20 g L^−1^ as carbon source) plates grown at 30°C for 5–7 days, by light microscope (Stereomicroscope System SZX10, Olympus, Hamburg, Germany). Colonies that were sporulating and producing high amounts of diffusible yellow pigment into the solid medium were designated as isolate BV365 and selected for further characterization. Genomic DNA of *S. anulatus* BV365 was extracted using GeneJET Genomic DNA Purification Kit (Thermo Fisher Scientific, United Kingdom). The full length 23S and 16S rRNA sequences from *S. anulatus* BV365 were deposited in GenBank under Accession numbers PP261333 and PP261334, respectively.

The appearance of colonies grown on MSF, MSM, ISP2, Sabouraud dextrose agar (SAB), tryptic soy broth agar (TSB), or Luria Bertani (LB) agar plates and colony morphology which included size, shape, texture, form, was observed after 3–5 days of incubation at 30°C using light microscopy. The morphology of isolate BV365 was observed using a field emission scanning electron microscopy (FESEM, Mira3 Tescan, Brno, Czech Republic) with an accelerating voltage of 10 kV. Prior to the observation, samples were fixed on carbon tape as support, sputter-coated with a thin layer of gold and recorded at various magnifications.

### 2.3 Production and preparation of *Streptomyces anulatus* BV365 culture extracts

A spore suspension of *S. anulatus* BV365 strain was prepared in 20% (v/v) glycerol, kept at −80°C, and used for the inoculation of cultures for pigment production experiments. The spore suspension was firstly inoculated into 20 mL medium. Medium MSF has been used as a source of carbon, starch, vitamins and minerals for microbial growth and production of biopigments. *S. anulatus* BV365 was incubated in an Erlenmeyer flask (1:5 culture to volume ratio) containing a coiled stainless steel spring for better aeration, at 30°C on a rotary shaker (constant mixing at 180 rpm) for 48 h. Upon incubation whole cultures (mycelium and supernatant) were extracted with ethyl acetate (1:1 ratio) by vigorous mixing at room temperature (60 min). The ethyl acetate fraction was separated by centrifugation (5,000 x g for 10 min at 25°C; Sorvall RC-5B Super Speed Centrifuge; Du Pont Instruments, United States). The ethyl acetate fraction was then dried over sodium sulfate (Na_2_SO_4_), filtered, and dried under reduced pressure at 40°C on BUCHI-Rotavapor R-300 (BÜCHI Labortechnik AG, Flawil, Switzerland) to give an orange powder.

The wavelength scan of the ethyl acetate crude cell extract was done from 200 to 700 nm using UV–Vis Spectrophotometer Ultrospec 3300pro (Amersham Biosciences, Piscataway, NJ, United States). The crude pigmented bacterial extract was used for further purification or activity tests.

### 2.4 Chromatographic separation of *Streptomyces anulatus* BV365 crude culture extract

Crude culture extracts were fractionated using flash chromatography on silica gel 60 (230–400 mesh; Merck, Darmstadt, Germany). The following solvent system was used for fractionation of approximately 500 mg of crude extract: n-hexane and ethyl acetate (7:3 ratio, 100 mL), ethyl acetate (250 mL) followed by ethyl acetate and methanol (7:3 ratio, 100 mL). The collected fractions were analyzed by thin-layer chromatography using aluminum-backed plates with a 0.25-mm silica layer (Kieselgel 60 F254; Merck, Darmstadt, Germany) and visualized by UV–vis spectral analysis. The fractions were collected based on the TLC analysis and determined Rf values, the organic phase was removed under reduced pressure, and fractions were weighted.

### 2.5 Metabolite analysis of *Streptomyces anulatus* BV365 using high-resolution LCMS/MS and GNPS

Dried extracts of separated fractions were dissolved in methanol and analyzed on a Bruker Impact II (Bruker Daltonics GmBH and Co. KG) instrument equipped with an electrospray ionization source (ESI, Apollo II), a hybrid quadrupole time-of-flight (qTOF MS) instrument, and coupled to an Elute (U) HPLC 1300 system. The ESI source was connected to an external pump (Hamilton syringe 2.5 mL) for pre-acquisition mass calibration using the Na-Formate calibrant solution (12.5 mL H_2_O, 12.5 mL isopropanol, 50 μL HCOOH conc., 250 μL NaOH 1M). Chromatographic separation was achieved on a Bruker Intensity Solo C18 (1.8 μm, 2.1 mm, 100 mm) column retained at 40°C and the mobile phase consisted of H_2_O containing 0.1% (v/v) formic acid (solvent A) and ACN (100%) containing 0.1% (v/v) formic acid (solvent B). The following gradient elution program for LC-qTOF HRMS was applied: 0–2 min: 95% A, 2–25 min: 95%–5% A, 25–28 min: 5% A, 28–30 min: 95% A with a flow rate of 0.3 mL/min. The Q-TOF HRMS method consisted of a full scan TOF survey (50–1,300 Da) and a maximum number of three DDA MS/MS scans. The source parameters were as follows: dry gas 8 L/min, nebulizer gas 1.8 bar, capillary voltage 4.5 kV and end plate voltage of 500 V. For the DDA MS/MS experiments, a collision energy (CE) ramp of 20–50 V was applied. Two μL of sample were injected. The instrument was controlled by Hystar and Otof software, while data processing was carried out using Data Analysis software version 6.0.

Acquired high-resolution LCMS/MS data was submitted to the Global Natural Products Social (GNPS) molecular networking tool (https://gnps.ucsd.edu) (Wang et al., 2016). Analysis was performed using default settings with a search performed against known library hits. Mirror images comparing LCMS fragmentation of acquired spectra with library hits were generated by GNPS.

^1^H and ^13^C nuclear magnetic resonance (NMR) spectra of the purified compounds were recorded using a Bruker Ascend 400 spectrometer (400 MHz, Bruker, United States) and their weight measured. Based on the yield of the pure compounds, productivity per L of culture was determined.

### 2.6 Cytotoxicity

Cytotoxicity of *S. anulatus* BV365 crude culture extract and purified fractions was assessed by 3-(4,5-dimethylthiazol-2-yl)-2,5- diphenyltetrazolium bromide (MTT) colorimetric assay with human fibroblasts (MRC5) and human colorectal carcinoma (HCT116) cell lines obtained from the American Type Culture Collection from (ATCC), as previously described (Hansen et al., 1989). Assays were carried out after 48 h of cell incubation in media (RPMI 1640 medium, Gibco™ by Thermo Fischer Scientific CE, supplemented with 10% fetal bovine serum (FBS), 100 U mL^−1^ penicillin and 100 μg L^−1^ streptomycin) containing test compounds at concentrations ranging from 100 ng mL^−1^ to 0.15 ng mL^−1^. The extent of MTT reduction to formazan within cells was measured by absorbance at 540 nm on Tecan Infinite 200 Pro multiplate reader (Tecan Group Ltd., Männedorf, Switzerland). The results are presented as a percentage of the control (cells treated with DMSO) that was arbitrarily set to 100%.

### 2.7 Antibacterial activity

#### 2.7.1 Antibacterial activity of crude extract and fractions

The antimicrobial activity of purified actinomycins and nonactin fractions against different strains of *Staphylococcus aureus* was determined using a previously reported 96-well microtiter plate assay (Casey et al., 2004). A dilution series of the purified pigment actinomycins and nonactin were prepared in dimethyl sulfoxide (DMSO). Controls containing solvent were carried out in each assay. The minimum inhibitory concentration (MIC) was determined as the lowest concentration of the compound at which no evidence of growth was observed.

#### 2.7.2 Antibacterial activity of dyed silk

The agar well diffusion method was applied to assess the antimicrobial activity of textile extracts (Nguyen et al., 2019). The threads taken from the dyed silk were flooded in LB medium (10 mg mL^−1^) and incubated at 37°C. After 72 h long mixing at 180 rpm, the suspensions were centrifuged for 10 min at 5,000 rpm (Eppendorf Centrifuge 5804R) and the supernatants sterilized with 0.22 μm filter (Millipore). The complete surface of LB agar plate was inoculated by spreading a volume of the microbial inoculum. Then, holes with a diameter of 6 mm were aseptically punched and filled with 150 µL of the textile extracts (10 mg mL^−1^). Thus, prepared agar plates were incubated at 37°C for 24 h. The growth inhibition was estimated by clear zones around holes. The textile extracts diffused into the agar medium and inhibited the growth of the tested microbial strains: *S. aureus* MRSA ATCC 43300, *S. aureus* ATCC 25923 and *S. aureus* 865.

Antibacterial activity of dyed silk, was also assessed in dynamic contact conditions, in the liquid LB culture on 2 microorganisms: *S. aureus* ATCC 25923 and *Escherichia coli* ATCC 10798. Overnight cultures of each test organisms were prepared in LB medium and diluted to a final optical density (OD_600_) of 0.1. The pieces of fabric samples (4 cm^2^, sterilized by autoclaving) were placed in the glass tubes containing 4 mL of each diluted bacterial test culture (OD_600_ = 0.1). Flasks were incubated at 37°C for 24 h with shaking and upon incubation the bacterial growth was estimated by measuring OD_600_ using UV–Vis Spectrophotometer Ultrospec3300pro (Amersham Biosciences, Piscataway, NJ, United States). Appropriate controls included medium with or without undyed fabrics and were included in each experiment. Tests were performed in triplicate.

### 2.8 Dyeing and washing of textile materials with *Streptomyces anulatus* BV365 purified actinomycins

The stock solution of fraction F3 (purified actinomycins) was prepared in acetone (20 g L^−1^) and further diluted with distilled water, so that final bath for dyeing contains either 0.5% or 1% o.w.f (on the weight of fabric) of pigment. The final dyeing system contained 10% of acetone and 90% of distilled water. Non-adjusted pH of a dyebath was pH 5. To optimize the procedure, dyeing was also performed at pH 3, pH 7 and pH 9. The pH was set using 0.01 M HCl or 0.1 M NaOH. The material-to-liquid ratio was 1:50. Dyeing was performed at different temperatures (25°C, 45°C, 65°C and 85°C) according to the scheme in Supplementary Figure S1 During total of 60 min. The kinetics of dyeing was investigated on a greige silk at 65°C under pH 5 of solution, during 10, 15, 30, 60 and 90 min.

The washing of samples was made according to standard ISO 105-C10:2006. The samples were washed in a bath containing 0.5% standard detergent at 40°C for 30 min. After washing, samples were rinsed thoroughly with distilled water and then with tap water and left to dry at room temperature.

The release kinetics of pigment from the silk was studied during 7 days in PBS at pH 7.4 using 100 mg of dyed silk in 50 mL of buffer solution. Temperature was maintained at 37.0°C by keeping the samples in water bath with shaking. The absorbance measurement was performed in the first day in the following intervals (15 min, 60 min, 2 h, 4 h, 6 h, 24 h) and after full 7 days (168 h).

### 2.9 SEM analysis of fibers before and after dyeing

Silk fibers surface morphology was investigated with scanning electron microscope (SEM) (JEOL 840A, Tokyo, Japan). The samples were coated with gold using a JFC 1100 device.

### 2.10 ATR-FTIR spectroscopy of isolated pigment and dyed silk fabric

Fourier transform infrared spectroscopy (FT-IR) was used to analyze the fibers’ surface chemistry using Nicolet™ iS™ 10 FT-IR (Thermo Fisher Scientific Inc., Waltham, MA, United States) spectrometer with Smart iTR™ Attenuated Total Reflectance (ATR) Sampling accessory. The spectra were recorded in the range of 4,000–600 cm^−1^ with 32 scans per spectrum.

### 2.11 UV-VIS measurements

The absorbance of *S. anulatus* BV365 F3 solution and the absorbance of the dyebaths before and after dyeing were recorded with a UV-VIS Spectrophotometer, Model: UV1800 (Shimadzu, Kyoto, Japan).

### 2.12 Color coordinates of dyed fabrics in CIELab space

The color coordinates and reflectance of samples were measured using SpectraflashSF350X (Datacolor, United States) under illuminant D65 using the 10° standard observer.

The reflectance of samples (R) was used to calculate of color strength (K/S value) at the minimum of the reflectance at 442 nm. Washing durability was tested after one and three cycles of washing by measuring color difference ΔE. Delta E (ΔE) is calculated as the mean difference between color parameters L, a and b which were measured before and after washing. ΔL* represents the difference in brightness, Δa* the difference on the red/green axis and Δb* on the yellow/blue axis in the Color Space Lab. All measurements were done in triplicate and expressed as average ±standard deviation (SD).

### 2.13 Irritation effect of dyed silk fabrics on 3D-reconstructed human epidermis model

The skin irritation test was done according to the modified OECD test guideline 439: “*In vitro* Skin irritation: Reconstructed Human Epidermis Test Method,” OECD Guidelines for the Testing of Chemicals, Section 4 Health effects (2021). This work was carried out following instructions detailed in standard procedure MIP016 – Procedure for Assessing Cell Viability using an MTT-Based Method, and Work Instruction WI-LSS-21010-BEL. All work was performed in a laboratory facility which holds current ISO9001:2015 Certification (Labskin, United Kingdom). Briefly, material discs (with and without pigment), white negative controls (4 discs soaked in PBS) and positive controls using 5% SDS (4 discs). The latter is known to reduce Labskin cell viability below 50% and to create an inflammatory response following 20 min of direct exposure. These control samples were compared to yellow dyed silk (4 discs for dyed silk). All Labskin units were exposed to the saturated control/dyed discs for 20 min at 37°C in an incubator, after that discs were removed, and Labskin1.1 was incubated at 37°C for an additional 24 h when the cytotoxicity assessment has been carried out. To obtain additional information about the irritation effect exerted by the test items, quantification of released IL-1α was carried out using ELISA. Experiments were done in triplicate.

### 2.14 Statistical analysis

Data handling, analysis and representation was carried out using Microsoft Excel 2016, GraphPad Prism8 and the Origin Pro 9.0 software. The data are expressed as mean ± SD.

## 3 Results

### 3.1 Isolation of orange pigment-producing bacterial BV365 isolate

**FIGURE 1:**
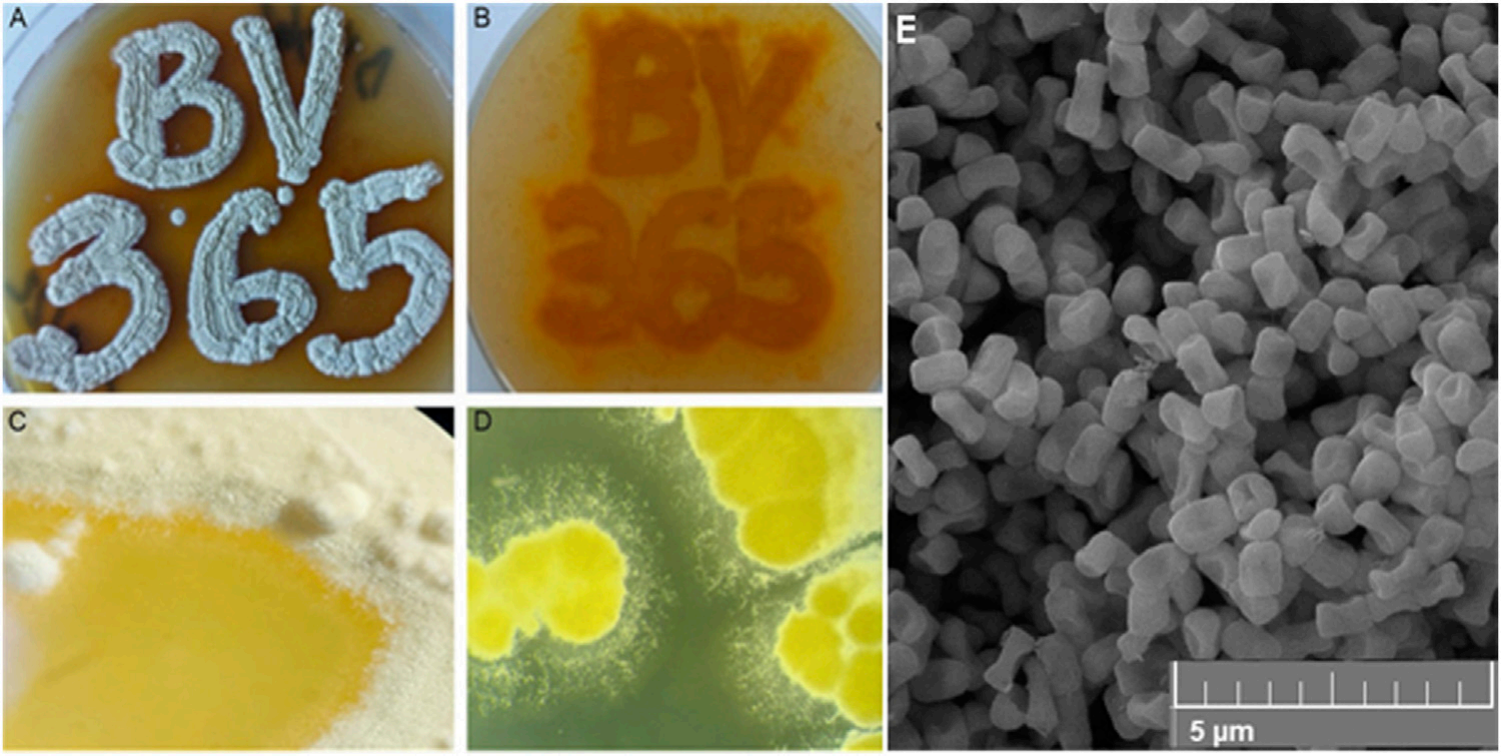
The appearance of pigmented *S. anulatus* BV365 grown on MSF **(A, B)** or TSB **(C, D)** solid media for 5 days at 30°C **(A)**. Scanning electron micrograph shows the spore morphology **(E).** Stereo microscope images, ×6.3 magnifications (System SZX10, Olympus), Field emission scanning electron microscopy (FESEM, Mira3 Tescan).

A number of bacterial strains were isolated previously from the ectomycorrhizosphere of black truffle (Djokic et al., 2011). Among them, an aerobic spore-forming strain with well-developed substrate mycelia and aerial mycelia that exhibited strikingly yellow to orange pigmentation on the solid media was designated as BV365 (Figure 1). Morphological studies showed that strain BV365 had the classic morphological properties of the genus *Streptomyces*. Further, sequence alignment using BLAST of the 16S and 23S rRNA regions found 100% sequence similarity to *S. anulatus*. Thus, we designated the newly isolated bacterium as *S. anulatus* BV365.

*Streptomyces anulatus* BV365 exhibited good growth with diffusible yellow to orange pigment production on all of the agar media tested after 5–7 days (Figures 1C, D; Supplementary Figure S2). The color of mature aerial mycelia was also yellow with evident liquid globules on top of the colonies. This isolate had the characteristic ability to sporulate abundantly on MSF and MSM media with spores being white to yellowish in color. Smooth spore surface morphology was observed under a scanning electron microscope (Figure 1E). The sporulation of *S. anulatus* BV365 on TSB and SAB agar plates was poor, whereas no sporulation was observed on ISP2 and LB agar plates. In this study, MSF agar stood out by the prominent stimulative impact on diffusible pigment production and was the best medium for *S. anulatus* BV365 sporulation (Figures 1A, B).

### 3.2 Fractionation and characterization of *Streptomyces anulatus* BV365 culture extract

In liquid media (shaking flasks), the production of yellow pigment commenced after 72 h and reached the maximal concentration at 120 h. A total of 1,000 mL fermentation broth was extracted using ethyl acetate and gave a crude extract with orange-yellow color (Supplementary Figure S3). The yield of the crude culture extracts from the MSF, TSB and ISP2 liquid media was 390, 150 and 190 mg L^−1^ respectively. The results confirmed previous conclusion that the soy flour supplemented with mannitol is favorable for the production of yellow pigment by strain *S. anulatus* BV365. The UV absorption spectrum of the ethyl acetate crude extract had defined maxima at 260, 420 and 440 nm (Supplementary Figure S3). The absorption maximum for the extract found between 400 and 450 nm, is typical of yellow to the orange spectral range.

TLC analysis of the ethyl acetate extract with ethyl acetate as a mobile phase showed four spots, which indicated the presence of four different compounds in our extract (Supplementary Figure S4). Therefore, four fractions were separated using flash chromatography, F1 to F4. Purified yields from 500 mg of crude culture extract were 25 mg (F1, 5%), 155 mg (F2, 31%), 215 mg (F3, 43%) and 72 mg (F4, 14.4%). Fraction F2 was subjected to further purification by precipitation with methanol, resulting in a white precipitate (94 mg) that was further characterized.

The HRESI-MS spectrum showed that the molecular weight of this precipitate was *m/z* 737.4484 [M + H]^+^ suggesting a molecular formula of C_40_H_64_O_12_. (calcd. 737.4476). The resulting material was sufficiently pure for NMR analysis. The ^1^H NMR and ^13^C NMR data agreed well with the nonactin data reported by (Wu and Sun, 2006; Saitô et al., 1988) (Supplementary Figures S5, S6). Therefore, this compound was unambiguously identified as nonactin and supporting NMR data is provided in the ESI (Supplementary Figure S9).

**FIGURE 2:**
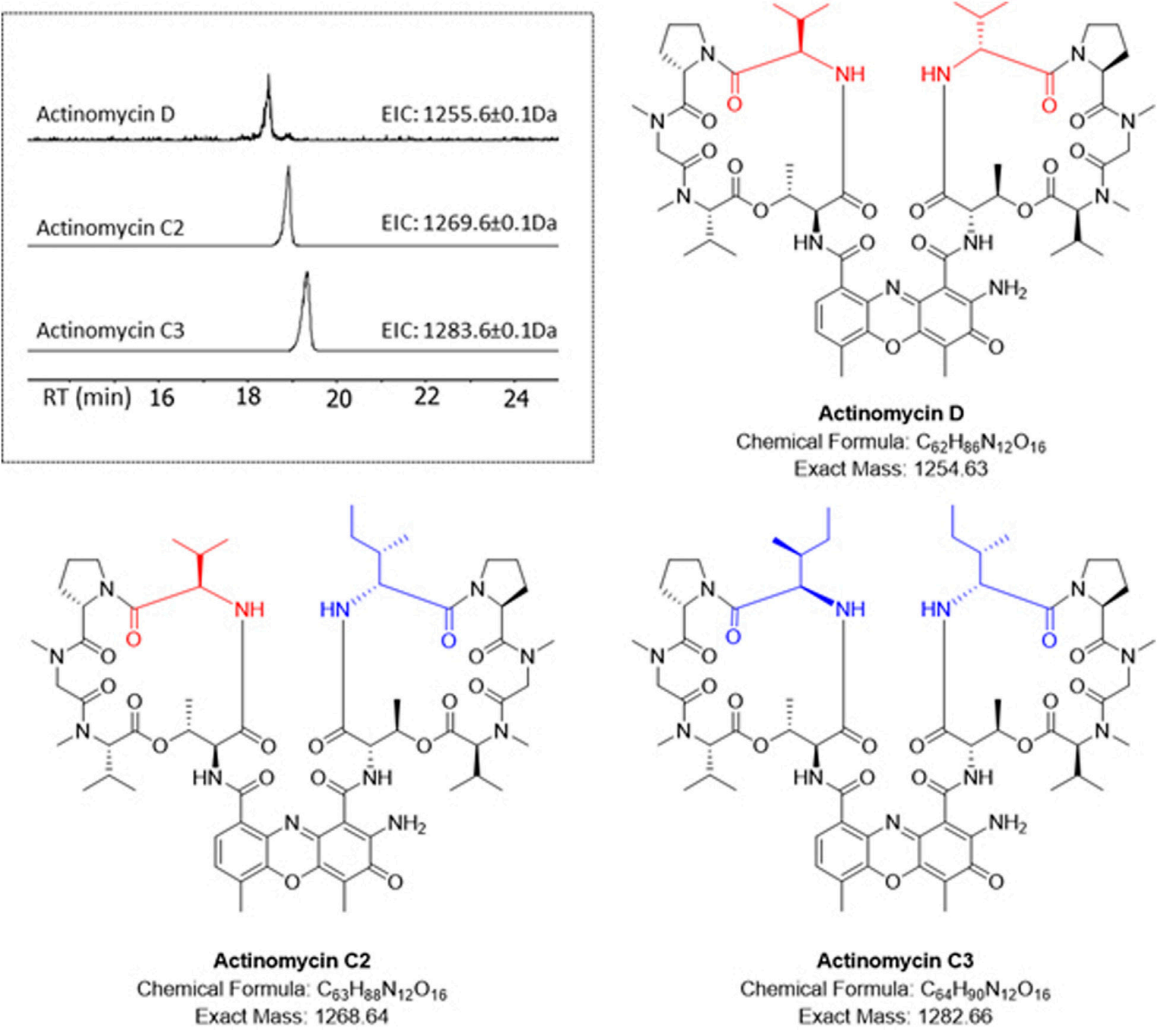
HR-LCMS/MS identification of the actinomycins in fraction F3. Different amino acid substitutions leading to various actinomycin structures can be observed with valine (red) or isoleucine (blue).

The HRESI-MS of fraction F3 showed the presence of a family of three highly related compounds (Figure 2). Comparison of the LCMS/MS data to the GNPS library enabled the identification of these compounds as members of the actinomycin family of natural products. The three members of the family included actinomycin D (*m/z* 1,255.6649 [M + H]^+^), actinomycin C2 (*m/z* 1,269.6526 [M + H]^+^) and actinomycin C3 (*m/z* 1,283.6703 [M + H]^+^) (Figure 2). All three molecules showed highly conserved MS/MS fragmentation patterns when compared to the corresponding library spectra (Supplementary Figure S7). NMR analysis was additionally performed for actinomycin D. The ^1^H NMR and ^13^C NMR data in comparison to those published by (Chen et al., 2012; Sarkar et al., 2008), corroborated the compound to be actinomycin D (Supplementary Figures S8, S9).

Overall, yields of commercially attractive actinomycin and nonactin were 167.7 mg L^−1^ and 73.32 mg L^−1^, respectively, making this strain unique novel producing strain of both compounds in single fermentation.

### 3.3 Cytotoxicity and antibacterial activity of pigmented *Streptomyces anulatus* BV365 crude and fractionated cell extract

The antibacterial activity was determined against a panel of Gram-positive *S. aureus* strains including MRSA (Table 1). *S. anulatus* BV365 crude extract as well as fractions containing nonactin (F2) and actinomycins (F3) showed strong antibacterial activities against the tested strains. F3 showed the highest activity with MIC values between 0.1 and 3 μg mL^−1^, while fraction with nonactin, also exhibited significant influence against drug-resistant forms of the pathogens *S. aureus*, with MIC values of 5–10 μg mL^−1^ (Table 1).

**TABLE 1:** Antibiotic and cytotoxic activities of crude culture extract of *S. anulatus* BV365, actinomycins (F3) and nonactin (F2) fractions presented as the MIC and IC50 values (µg mL^−1^) values against MRC5 and HCT116 cells.

	*S. aureus* ATCC 29231	*S. aureus* ATCC 25923	*S. aureus* MRSA ATCC 43300	*S. aureus* NCTC 6571	*B. cereus* ATCC 14579	MRC5	HCT116
Crude culture extract	0.125	2.000	1.000	1.000	0.250	0.025	0.030
Actinomycins fraction (F3)	0.150	0.150	0.300	0.150	0.100	0.015	0.020
Nonactin fraction (F2)	5.000	5.000	10.000	8.000	0.250	0.003	0.008

The cytotoxic antiproliferative effect was also determined on the human fibroblasts cell line (MRC5) and human colorectal carcinoma cell line (HCT116) (Table 1). The crude cell extract exhibited a high cytotoxic effect on MRC5 cells with IC_50_ value at a concentration of 25 ng mL^−1^ and caused 95% cell death at a concentration of 50 ng mL^−1^. Both fractions, F2 containing nonactin and F3 containing actinomycins showed remarkable cytotoxic activity against the tested human MRC5 cells with IC50 values 3 of and 15 ng mL^−1^, respectively, with F2 being 2.5- to 5-fold more cytotoxic.

### 3.4 Dyeing with actinomycins and material properties

Actinomycins from *S. anulatus* BV365 were used for dyeing multifiber fabric, and dyeing procedure was further optimized in terms of investigating the kinetics of dyeing on silk fabric. In Supplementary Figure S10 the UV-vis spectrum of the dyebath is presented. The major peak was detected at 264 nm, while a much smaller peak in the visible area was detected at 442.5 nm. The peak at 442.5 nm in the visible part of the spectrum was used to follow the exhaustion of the dyebath after dyeing. To study the coloring properties of the actinomycin fraction, the dyeing procedure on multifiber fabric style 49 was performed at 65°C and 85°C using different pHs of the dye solution. The quick washing test was also performed after dyeing, comprising one cycle of washing at 40°C, to determine washing fastness of dyed materials.

**FIGURE 3:**
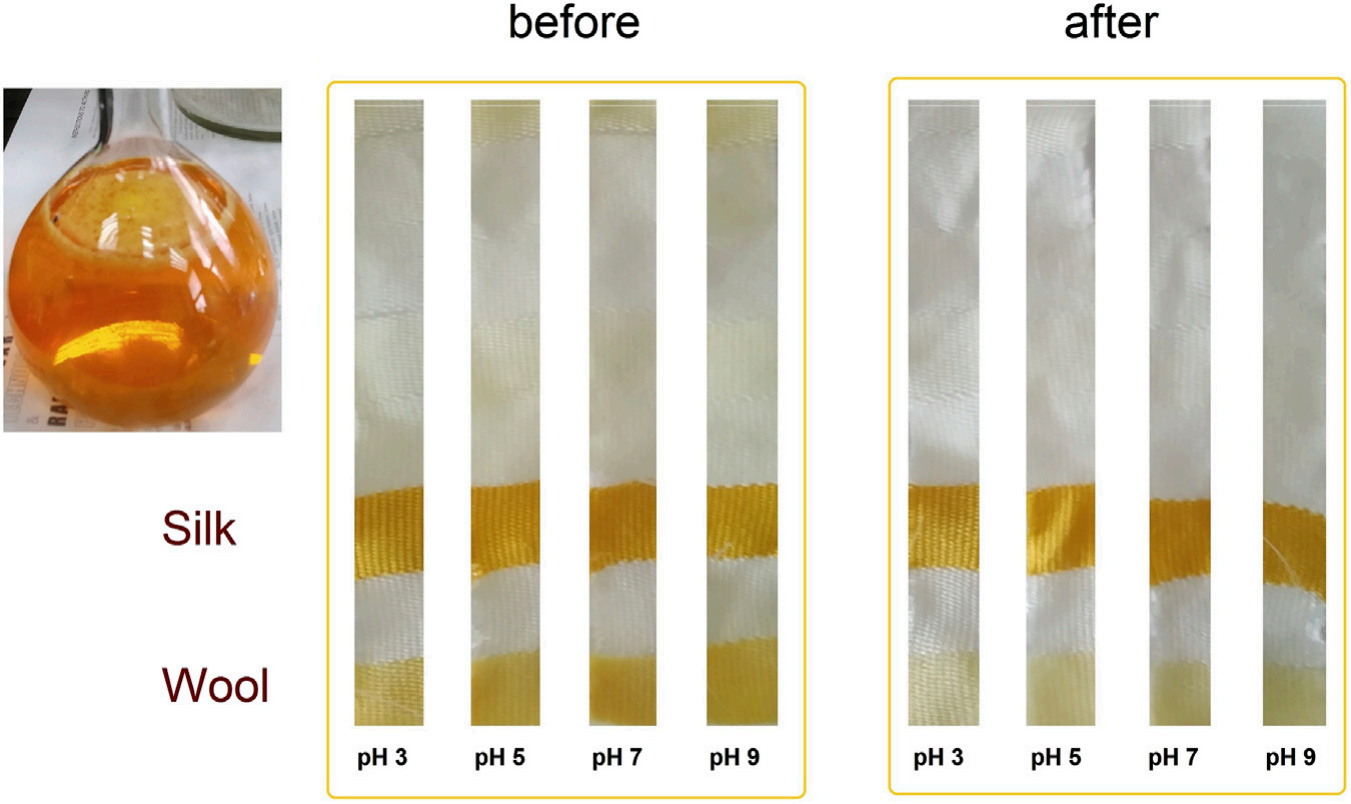
Photograph of the actinomycins fraction F3 in a solvent system consisting of acetone:water (10:90) and photographs of samples of multifiber fabric dyed at 65°C, taken before and after washing.

**FIGURE 4:**
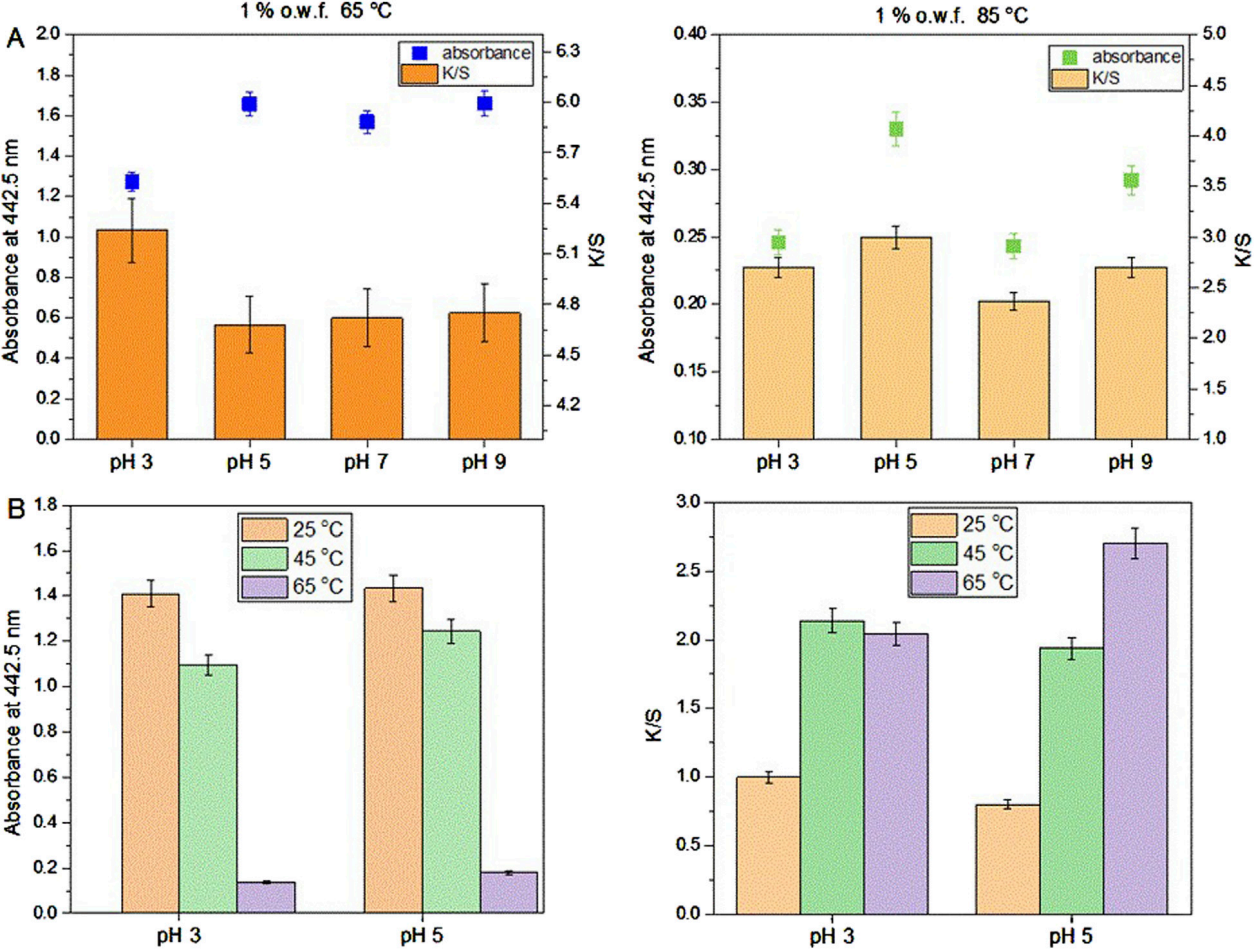
Dyeing of multifiber fabric, results obtained for silk part at different pH and temperature using 1% o.w.f. in the dyebath **(A)**; Absorbance of the dyebath after dyeing of silk on multifiber fabric (left) and K/S values of silk part dyed at 25, 45°C and 65°C and at pH 3 and pH 5 of the dyebath **(B)**.

After preliminary experiments of dyeing with the actinomycins fraction F3 it was evident that the dying process performed on multifiber fabric is very selective and only effective for wool and silk fibers, i.e., protein fibers (Figure 3). A preliminary washing fastness test was also performed with a warm detergent solution and was evident that the dyed silk fibers have good color fastness to washing as the color only slightly changed (Figure 3). To quantify the changes after the dyeing at different pH, we observed the decrease of the absorbance of the dyebath after dyeing and the change of color intensity estimated through the K/S value derived from the reflectance measurements on the fabric (Figure 4A; Supplementary Table S1). This evidenced that much higher K/S values are obtained for dyeing at 65°C compared to 85°C. On the other hand, the absorbance decrease in the dyebath was much greater at 85°C despite the fact that color is not more intensive on this set of multifiber fabric samples. This suggests that actinomycins are unstable at higher temperatures. Therefore, for further experiments, temperatures of 65°C or lower were chosen. Since silk is a protein filament, and higher K/S is obtained at more acidic pH of the dyebath, for further investigation, pH 3 and pH 5 were used at three different temperatures 25°C, 45°C, and 65°C. Furthermore, this initial experiment revealed that the absorbance of the residual dyebath was still very high (>1) (Figure 4B). Consequently, further investigation should proceed with a lower concentration of the dyebath, 0.5% o.w.f.

**FIGURE 5:**
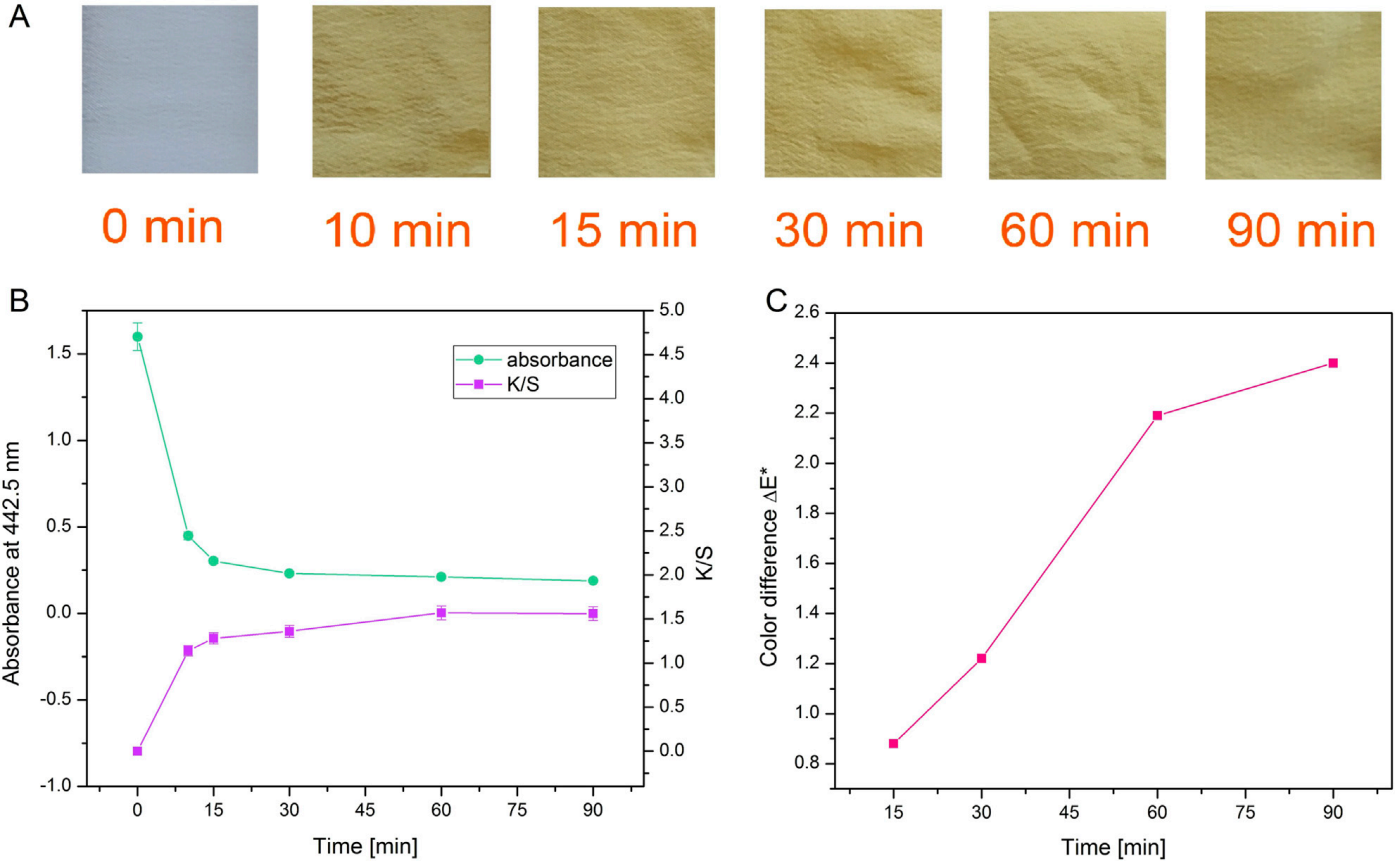
Photographs of silk after dyeing for different durations with actinomycins fraction F3 **(A)**. Absorbance of the dyebath and corresponding color strength of silk fabric dyed during different times **(B)** and color difference ΔE* between samples dyed during various times and sample dyed for 10 min **(C)**.

As can be seen in Figure 4B, dyeing at 25°C led to the lowest dyebath exhaustion and also the lowest K/S value of the dyed fabrics. The most optimal condition for dyeing was 65°C at pH 5. This was further supported by the fact that pH 5 is the pH of the freshly prepared F3 solution, therefore additional adjustment of the pH of the dyebath is unnecessary, which bears a positive economic impact. Since it was established that using 0.5% owf, pH 5 of dye solution and 65 °C temperature during dyeing, the kinetics of dyeing was tested under these conditions. Kinetic experiments were performed on white silk fabric (Figure 5A; Supplementary Figure S11) and after dyeing it was shown that the actinomycins exhibit high affinity and high specificity towards silk, more than towards the wool. The absorbance of the dyebath decreases strongly in the first 10 min of the dyeing of silk, and after 30 min reaches equilibrium (Figure 5B).

When a decrease of absorbance and an increase of K/S (Figure 5B; Supplementary Table S1) are presented together, it is obvious that exhaustion of the dyebath is strongly correlated with the intensity of the color on the material which is expressed through K/S value. It is worth noting that even after 10 min of dyeing, there is a great exhaustion of dyebath and good color strength. Moreover, by observing the values of K/S it can be concluded that for the greatest color strength, dyeing should be performed for 60 min. The color difference was calculated for all samples compared to the sample dyed for 10 min. The biggest difference and slope of the curve occurred when dyeing was performed for 60 min (Figure 5C).

Washing fastness results (Supplementary Figure S12) revealed that after washing color difference ΔE* is increasing, and the sample becomes less red (decrease of a* coordinate) and less yellow (decrease of b* coordinate). Lightness of samples L* is almost unchanged after washing. Considering the low standard deviation of color strength in the samples, we can conclude that coloration evenness is good and satisfactory.

**FIGURE 6:**
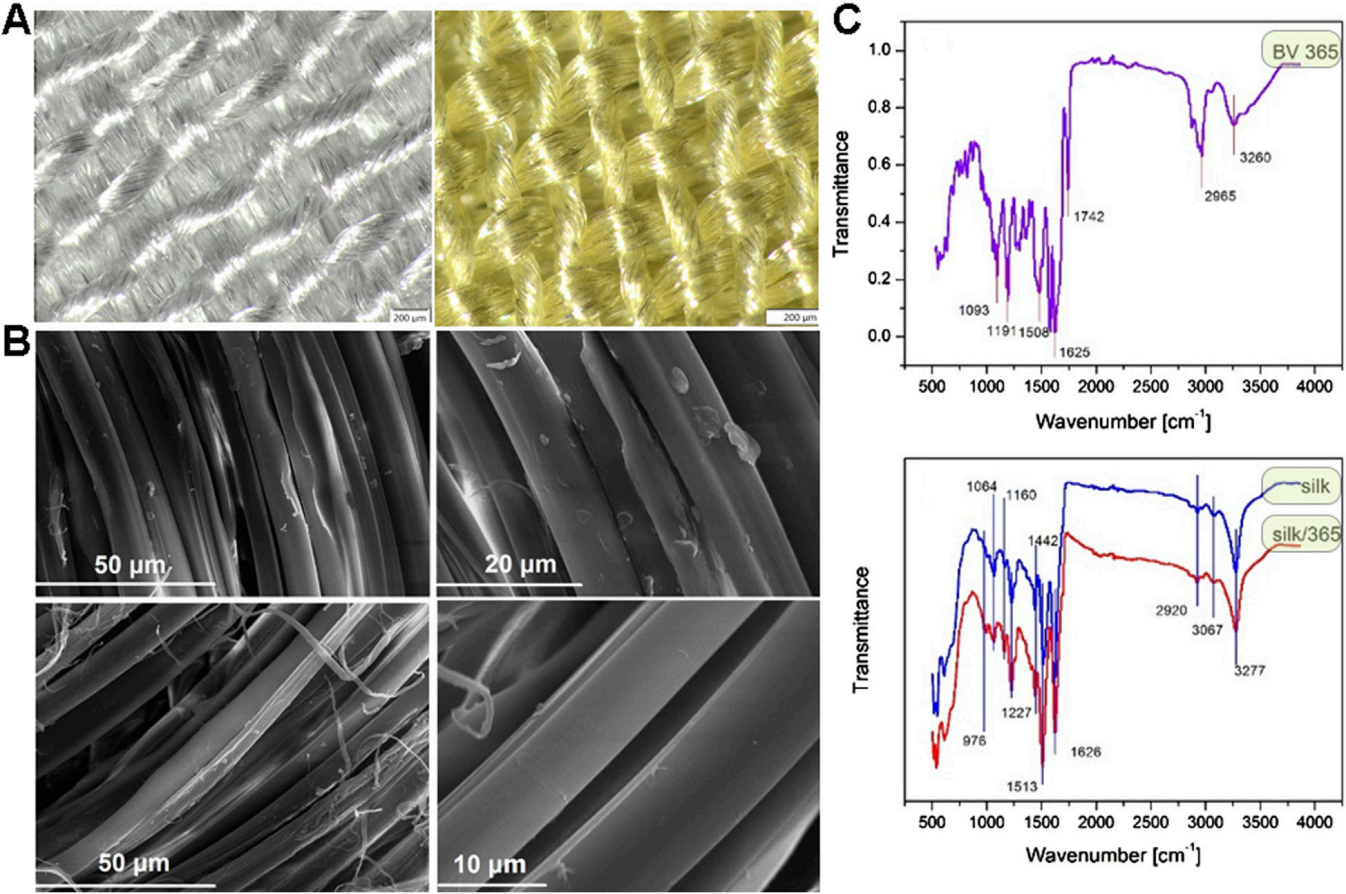
Stereo light microscope images of untreated sample of silk (left) and dyed silk (right) at 6,3× magnification **(A)**; SEM images of untreated sample of silk at different magnifications (top) and dyed silk at different magnifications (bottom) **(B)**. ATR-FTIR spectra of *S. anulatus* BV365 pigment actinomycins (top) and silk fabric before and after dyeing with actinomycins containing F3 (bottom) **(C)**.

The stereo light microscope and SEM analyses of samples after dyeing with actinomycins fraction F3 do not show any significant changes in fibers morphology (Figure 6).

ATR-FTIR of actinomycins used for dyeing revealed several major peaks (Figure 6C top), at 3,260, 2,965, 1742, 1,625, 1,508, 1,191 and 1,093 cm^−1^. These peaks were assigned to amide NH stretching (3,260 cm^-1^), methyl CH asymmetric stretching (2,965 cm^−1^), carbonyl (1740–1,640 cm^−1^), aromatic ring (1,508 cm^−1^), C-N stretching (1,191–1,093 cm^−1^). These peaks are in line with those reported in the literature for actinomycins isolated from different bacterial strains (Chandrakar and Gupta, 2019; Mangrolia and Osborne, 2020; Shetty et al., 2014). ATR-FTIR analysis was performed to study the possible interaction between silk and actinomycins fraction F3 (Figure 6C bottom).

Typical peaks for silk were recorded, 3,277 cm^−1^and 3,067 cm^−1^, corresponding to NH stretching in amide and CNH overtone respectively; 2,920 cm^−1^ corresponds to asymmetric CH stretching, 1,626 cm^−1^ C = O stretching (Amide I of fibroin), 1,513 cm^−1^ NH out-of-phase (specific for fibroin as well), 1,442 cm^-1^CH in plane bending vibration, 1,227 cm^−1^C-N stretch and C-C stretching (Amide III), 1,160 cm^−1^C-N stretching in tyrosine, 1,064 cm^−1^C-C stretching β-sheet, 976 cm^−1^ CH3 rocking. After dyeing, the peak at 1,626 cm^−1^ which corresponds to carbonyl group is slightly shifted toward higher wavenumber (1,630 cm^−1^) and decreased in intensity; In addition, there is a significant increase in intensity of the bands at 1,513, 1,227, 1,160, and 1,064 cm^−1^. This suggests that the pigments bind to the silk through the interaction with its functional groups and most probably through interaction with the carbonyl group in silk fibroin. According to FTIR, and taking into account the structure of silk protein and actinomycin in F3, we can assume that two types of possible interactions are present, the ionic interactions between carbonyl group in silk and primary or secondary amine from actinomycin, which can results in a Schiff base formation, and the van der Walls interaction i.e., formation of hydrogen bonds between secondary amines in actinomycin and carbonyl groups. The Schiff base formation in this case may be indicated by this shift of carbonyl groups peak.

In order to confirm the permanent bonding of pigment to silk, we performed experiments and measured release kinetics of dyed silk fabric up to 7 days of keeping the fabric at 37°C in PBS (Supplementary Figure S13). After 7 days in PBS there was not any peak present and detected in UV-VIS and sample remained colored which means that binding between silk and pigment is permanent, and most probably the consequence of Schiff base formation, due to abundance of amino and carbonyl groups.

### 3.5 Evaluation of skin irritation of dyed silk fabrics in 3D-reconstructed human epidermis model

**FIGURE 7:**
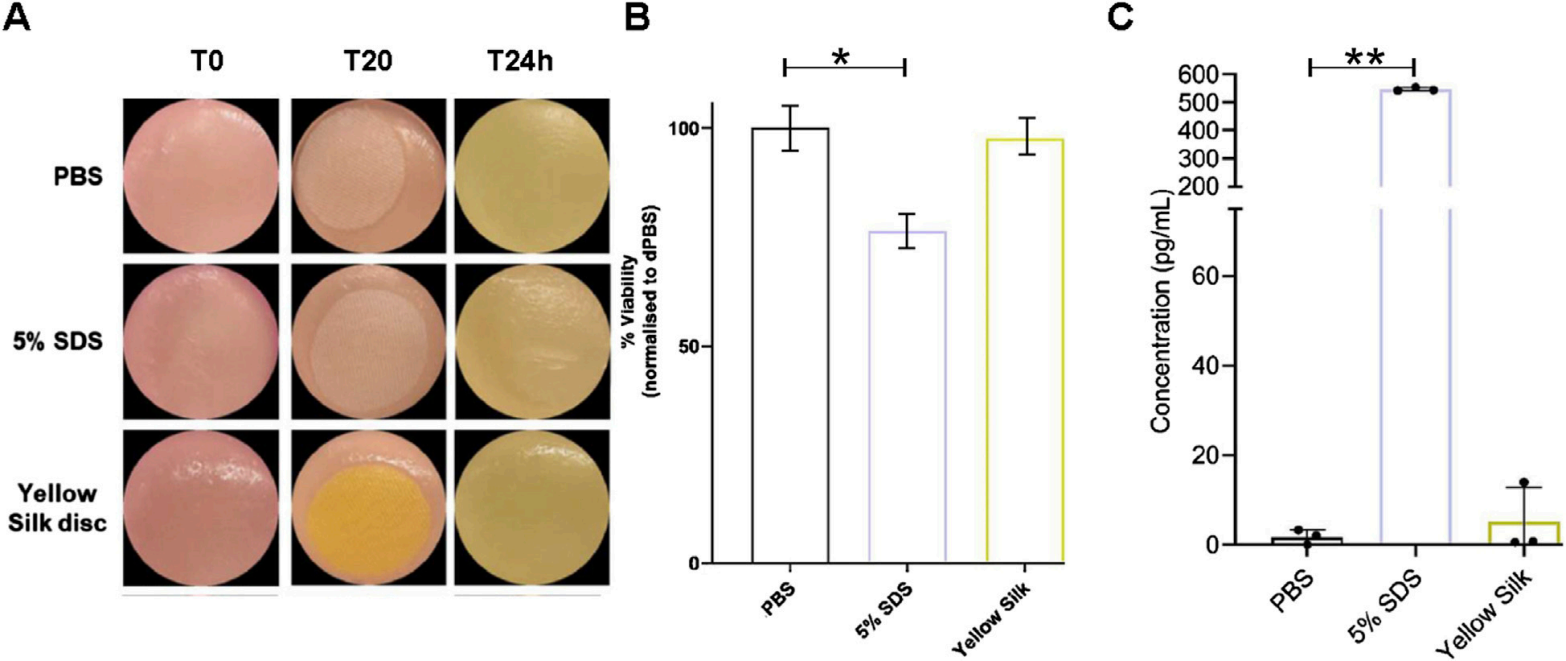
Macroscopic appearance of Labskin1.1 model **(A)**. Cell viability was calculated as % of MTT reduction to formazan compared to negative control treated with PBS **(B)**. Quantification of release of proinflammatory cytokine IL-1α after treatment **(C)**. Results are averages of n = 3, error bars represent SD and values were compared to the PBS-treated control using a t-test; (*p ≤ 0.01, **p ≤ 0.01) (* = *p* < 0.01; ** = *p* < 0.001).

The skin model used in the skin irritation tests, Labskin 1.1 (LABSKIN, United Kingdom), is a commercially accessible reconstructed human epidermis model prepared from human keratinocytes. The model was developed to be a multifaceted and fully differentiated human keratinocyte. The cytotoxicity of dyed silk fabrics was assessed by quantification of cell viability in Labskin 1.1 units using an MTT assay. Pieces of silk were transferred onto Labskin1.1 units (Figure 7A).

The results of the MTT assay are shown in (Figure 7B). When compared to the negative control silk (soaked in PBS), Labskin exposed to a positive control silk (soaked in 5% SDS) had the cell viability reduced by 23.77%, while Labskin reduction of cell viability exposed to the actinomycin dyed silk was only 2.46%. However, the experiments further demonstrated that SDS-soaked silk created a strong inflammatory response while actinomycins dyed silk did not cause a significant inflammatory effect (Figure 7C). Pro-inflammatory cytokine IL-1α is known to be produced in human skin after exposure to deleterious chemicals. The amount of IL-1α in the undernatant correlates with the viability of the cell tissue. The Labskin exposed to 5% SDS (known irritant) for 20 min, released approximately 308 times more IL-1α than the PBS control while the Labskin exposed to dyed silk for 20 min, released about 2.9 times more IL-1α than the PBS control (Figure 7C). Collectively, these experiments indicated that silk dyed with actinomycins has good biocompatibility with reconstructed human epidermis without exerting any significant cytotoxic effects on keratinocytes phenotype or functions.

### 3.6 Antibacterial activity of the dyed silk fabrics

In addition to biocompatibility, antibacterial activity is added significant factor influencing usage of any material for industrial and medical purposes. Antibacterial activity of silk fabrics, dyed with actinomycins fraction for 10, 15, 30, 60 and 90 min, before and after one and three cycles of washing was tested against *S. aureus* MRSA ATCC 43300, *S. aureus* ATCC 25923, clinical isolate *S. aureus* 865 and *E. coli* ATCC 10798 using an agar well diffusion method (Supplementary Table S2). In this case, silk fabrics were found to be efficient against *S. aureus* strains, whilst no inhibition was observed against *E. coli*. The results of the tests against *S. aureus* strains showed that silk dyed for 15 min possessed maximum antimicrobial activity against *S. aureus* strains, including *S. aureus* MRSA. Also, the results indicated that silk dyed with the fraction F3 retains antibiotic activity even after the third wash.

The antibacterial activity of dyed silk was also assessed using a standard test method for revealing the antimicrobial activity of immobilized antimicrobial agents under dynamic contact conditions (ASTM E2149–01). This method is intended to assess the resistance of antimicrobial-treated samples to microbial growth under dynamic contact conditions, in liquid LB culture on 2 microorganisms: *S. aureus* ATCC 25923 and *E. coli* ATCC 10798. The results of antimicrobial activity showed that silk samples dyed during 10, 15, 30, 60 and 90 min, ensured maximum reduction of tested *S. aureus* strain, whereas did not provide any antibacterial activity against *E.coli* 10,798 (Supplementary Figure S14). These results confirmed that a 10-min dyeing of silk fabrics with actinomycins fraction is sufficient to achieve maximum antimicrobial functionality, implying that such silk could be viable medical textile materials for the use in healthcare sector. On the other hand, 60-min dyeing of silk ensures the washing fastness of the dyed silk and durability of functionalization, while high antimicrobial activity is preserved.

## 4 Discussion

*Streptomyces anulatus* BV365, isolated from ectomycorrhizosphere soil of the black truffle, morphologically apparent and with colored mycelia, was selected for its competence to efficiently produce yellow to orange pigment on different solid media (Supplementary Figure S2). The black truffle is known to support a variety of both fungal and bacterial communities (Antony-Babu et al., 2014; Herrero de Aza et al., 2022) but their exact roles and functional potential still have to be determined. We herein have shown that *S. anulatus* BV365 produces two bioactive principles that can be purified upon chromatographic separation. NMR and HR-MS/MS analysis indicated the compounds to be nonactin and the three actinomycin analogues actinomycin D, C2 and C3.

Actinomycin D is an antibiotic and anticancer compound discovered in 1940 from *Actinomyces antibioticus* (Waksman and Woodruff, 1940), and since then, more than 30 actinomycins have been discovered from natural sources (Wang et al., 2017). Until now, there are various *Streptomyces* species identified to be capable of producing actinomycins. *Streptomyces heliomycini* and *Streptomyces* sp. MS449 (Chen et al., 2012) simultaneously produce actinomycins D, X0β and X2, with fermentation titers of actinomycin D at 458 and 1770 mg L^−1^, respectively (Wang et al., 2017). When the culture *S. anulatus* BV365 was grown in liquid MSF medium in the flask, 167,7 mg L^−1^ of actinomycins was obtained, which is in line with the literature reports on bacterial production of actinomycin D.

*Streptomyces* bacteria are producers of macrocyclic polyether ionophores such as nonactin (Matarrita-Carranza et al., 2021; Zhan and Zheng, 2016; Rawangkan et al., 2022). Nonactin has the ability to transport cations (alkali metal and NH4^+^ ions) through synthetic and biological membranes, and also shows antimicrobial and anticancer activities (Kusche et al., 2009; Lee et al., 2010; Žižka, 1998). Nonactin is used for the preparation of ion-selective electrodes and sensors (Joly et al., 2022; Karakuş and Pekyardιmcι, 2006). The ammonium ion sensor has potential function in the assessment of ammonium and ammonia in environmental contamination control, clinical analyses and other industry applications (Joly et al., 2022).

Both actinomycins and nonactin isolated from *S. anulatus* BV365 exhibited very strong cytotoxic activity against the human fibroblasts cell line (MRC5) and colon cancer cell line (HCT116) with IC50 values of 15 ng mL^−1^ and 20 ng mL^−1^ respectively, for actinomycins, and 3 ng L^−1^ and 8 ng L^−1^ respectively, for nonactin. Indeed, actinomycin D has been widely used in clinical practice since 1954 as an anticancer drug to treat tumors, such as Wilms and Ewing tumors, sarcomas, and choriocarcinoma (Falk Delgado et al., 2019; Gamboa et al., 2020; Hoang et al., 2018). It was the first antibiotic demonstrated to have anticancer activity. Actinomycin D binds to single- and double-stranded DNA and also inhibits RNA polymerase by complexing with DNA via deoxyguanosine residues (Goftar et al., 2014; Karpiński and Adamczak, 2018).

There is significant interest in substituting synthetic dyes in the textile industry with eco-friendly alternatives. Bio-based pigments derived from various plant species, fungi, bacteria, and algae have garnered attention as sustainable coloring agents for textiles (Periyasamy, 2022; Muthusamy et al., 2020). Microbial pigments, emerging as a renewable source, have been particularly distinguished for achieving durable and vibrant coloration (Mandal et al., 2017; Nuanjohn et al., 2023; Ren et al., 2018). While termed bacterial pigments, these metabolites function more like to dyes rather than pigments in textile science despite their insolubility in water, due to their chemical bonding with textile materials (Kramar and Kostic, 2022). Previously, Chen et al. demonstrated the application of similar actinomycin X2 from marine-derived *Streptomyces cyaneofuscatus*, in the dyeing and finishing of silk fabric (Chen et al., 2021). Furthermore, Zhou et al. reported immobilization of actinomycin-X2 onto a prepared silk fibroin (Zhou et al., 2023b). In this study, after obtaining high quantities of pigmented crude culture extracts from the new strain *S. anulatus* BV365, after a single chromatography step, we obtained fraction enriched with actionomycins that was suitable for further dyeing study. We have shown that it can be used as an efficient dye for silk fibers to very deep shade, and wool fibers to a noticeable, but much lower shade depth. Dyeing of textile materials whether in the form of fibers, yarn, or fabrics, follows the established process consisting of several phases (Grishanov, 2011). First, the dye molecules from the solution are absorbed onto the fiber surface, then dye molecules diffuse from the fiber surface into the fiber interior, which is usually governed by fiber swelling and can be facilitated by increasing the temperature during dyeing. In the final phase, dye molecules form bonds with functional groups of fiber, and the type of bonds formed in this phase ensures the washing fastness. From our results, the following can be concluded. Since the main antimicrobial activity of functionalized silk comes from the actinomycins fraction F3, using the short dyeing times enables high diffusion activity precisely because actinomycins did not form permanent bonds with silk. In this case they are absorbed into silk fiber, but also release relatively easily. This allows the actinomycins to diffuse from the fiber into the medium with bacteria and provide both a wide zone of inhibition (Supplementary Table S2) and high antibacterial activity in dynamic contact mode (Supplementary Figure S13). On the other hand, dyeing for a longer time enables the formation of bonds between silk and actinomycins as seen also using FTIR (Figure 6C) which apparently reduces the diffusion of actinomycins towards medium containing bacteria, but still provides excellent antibacterial activity as seen with static diffusion test and dynamic contact test. In terms of durability of the functionalization, longer times are preferable over the short ones for preservation of materials’ functionality.

Silk, with its longstanding use in medicine, particularly in delicate surgeries like eye procedures, owes its popularity to biocompatibility and strong mechanical properties (Holland et al., 2019). However, there are reports of adverse reactions, especially in silk sutures containing trace amounts of sericin urging comprehensive biocompatibility testing. Additionally, silk found application in surgical scaffolds for abdominal surgeries and textile materials in dermatology, effectively treating conditions like atopic dermatitis, thanks to its non-irritating surface, especially when combined with antimicrobial agents (Holland et al., 2019).

Skin irritation commonly occurs as an inflammatory response triggered by contact with the skin with keratinocytes playing a key role in regulating these inflammatory reactions. Application of an irritant leads to increased production and release of Interleukin 1 alpha (IL-1α) from epidermal keratinocytes (Coquette et al., 2003). The extracellular release of IL-1α is widely recognized as an indicator of chemical irritants (Mullerdecker et al., 1994; Corsini et al., 1999; Perkins et al., 1999; Kidd et al., 2007). To evaluate the biocompatibility of the tested fabrics skin irritation tests were performed. The results showed that Labskin exposed to the dyed silk caused a weak cytotoxicity and inflammatory response. In addition, silk dyed with the actinomycins fraction F3 retains promising antibacterial activity *in vitro* against *S. aureus* strains even after the third wash.

Taken together findings from this study provide evidence for the medical application of silk functionalized with actinomycins for future development of anticancer surgical threads or wound scaffolds for post-cancer surgery treatment.

## 5 Conclusion

This study investigated synchronized coloration and functionalization of silk fabric with actinomycins produced by fermentation of the new bacterial isolate *S. anulatus* BV365, collected from ectomycorhizosphere of the black truffle. An antimicrobial and antitumor family of chromogenic lactone peptides, actinomycin D, C2 and C3, together with the polyether ionophores, nonactin, were produced in high yields through the fermentation. This bioprocess can be further optimized by intensifying fermentation conditions, while more efficient and greener extraction procedures could also be applied. Compounds exhibited significant cytotoxicity against a human cell lines MRC5 and HCT116 and showed strong antibacterial activity against different *S. aureus* strains including MRSA. Actinomycins were used for silk dyeing and the efficient and stable functionalization of the material for biomedical purposes. The physical properties of dyed silk were analyzed by FTIR and SEM, the results suggest that the pigments bind irreversibly to the silk through the interaction with the carbonyl group in silk fibroin. Therefore, other materials and surfaces containing free carbonyl groups should be considered in future for the similar functionalization. Considering its excellent antibacterial activity, biocompatibility and low immune response, silk dyed with actinomycins can be regarded as one of the promising candidate for future applications in medicine and healthcare.

## Data Availability

The datasets presented in this study can be found in online repositories. The names of the repository/repositories and accession number(s) can be found below. https://www.ncbi.nlm.nih.gov/genbank/, PP261333 https://www.ncbi.nlm.nih.gov/genbank/, PP261334.
