# Supplementary material for: Functionalization of silk with actinomycins from Streptomyces anulatus BV365 for biomedical applications
Source: Front Bioeng Biotechnol. 2024 Sep 19;12:1466757. doi: 10.3389/fbioe.2024.1466757 (PMC11447452; doi:10.3389/fbioe.2024.1466757)
Supplement: Supplementary file 1 [file DataSheet1.docx]

Supplementary Material

#
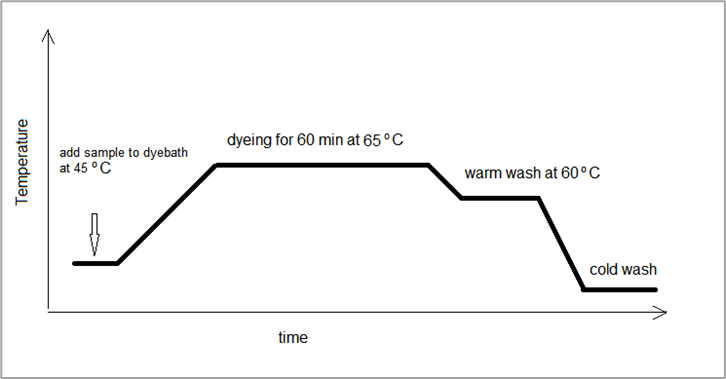


# Figure S1. Schematic diagram of the procedure for greige silk dyeing at 65 °C.


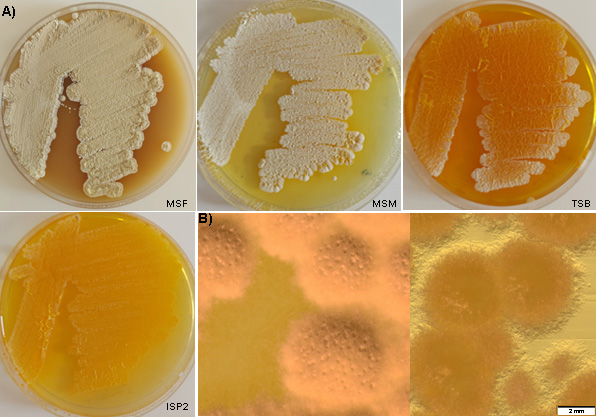


# Figure S2. (A) The appearance of pigmented *Streptomyces anulatus* BV365 grown on MSF, MSM, TSB and ISP2 solid media for 5-7 days at 30 ^o^C. (B) Stereomicrographs show representative colonies of *Streptomyces anulatus* BV365. Diffusible yellow pigment was produced on several tested media. 5x magnifications (SystemSZX10, Olympus, Hamburg, Germany) Bars: 2 mm.


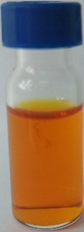

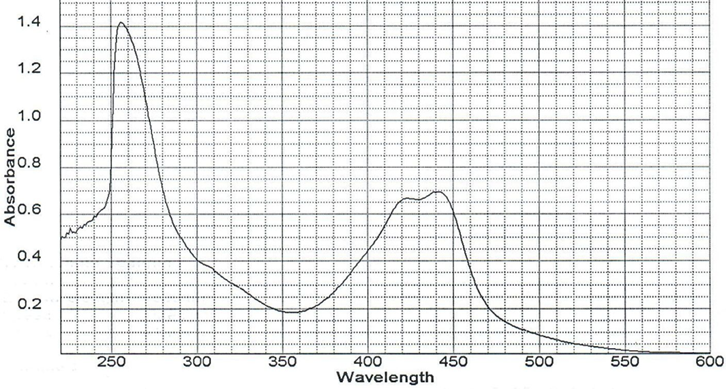


**(A)**

**(B)**

# Figure S3. (A) Ethyl acetate extract of the liquid culture; (B) UV-Vis spectrum of the crude ethyl acetate extract of the complete bacterial culture (UV–Vis Spectophotometer Ultrospec 3300pro (Amersham Biosciences).

#
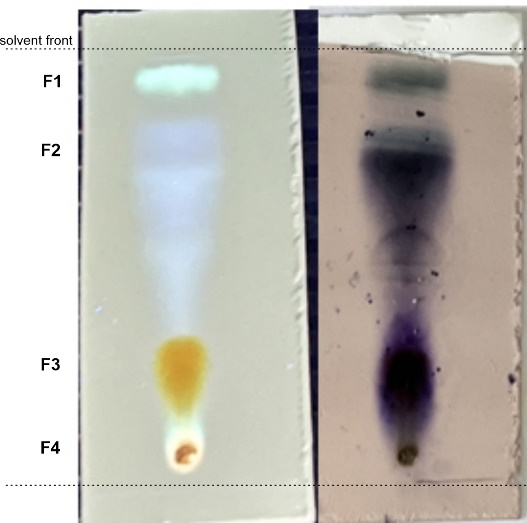


# Figure S4. Thin layer chromatography (TLC) of *Streptomyces anulatus* BV365 crude culture extract (eluent 100% ethyl acetate). Crude ethyl acetate extract comprises 4 separate fractions as revealed from its TLC chromatogram designated as F1, F2, F3 and F4 which showed 4 major spots with Rf values of 0.92, 0.72, 0.38 and 0.03. Right - visualization under ultraviolet light at 366 nm, left – staining with *p*-anisaldehyde stain.

#
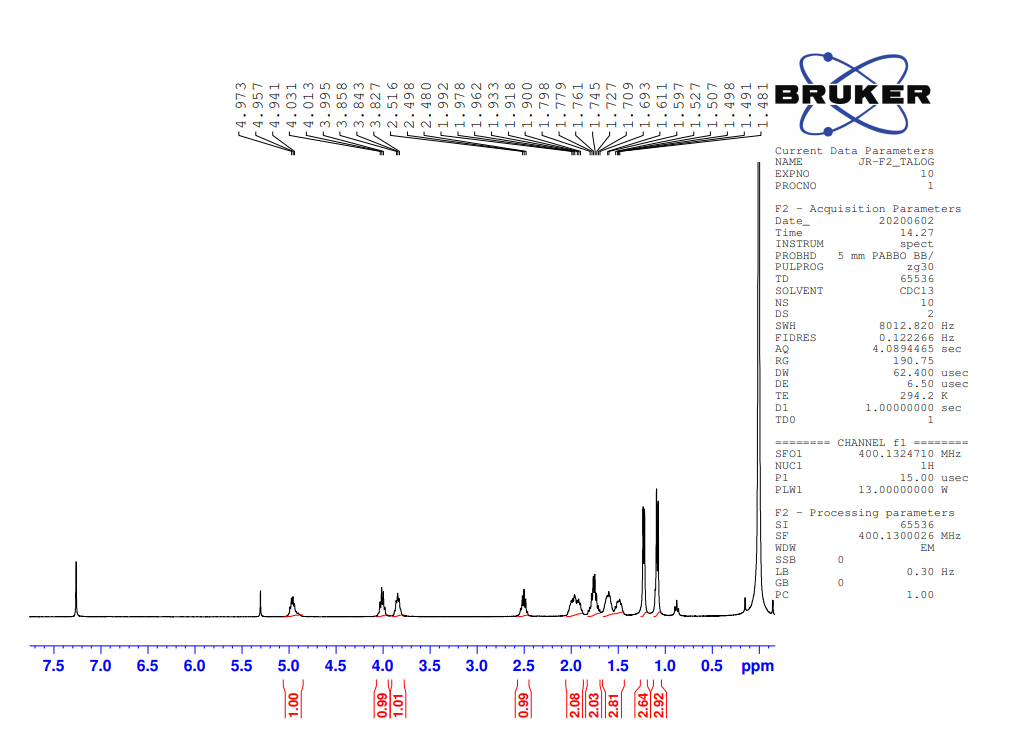


# Figure S5. ^1^H spectrum of Nonactin (F2-precipitate). ^1^H NMR (400 MHz, CDCl_3_) δ 5.03 – 4.84 (m,4H), 4.12 – 3.92 (m, 4H), 3.90 – 3.73 (m, 4H), 2.60 – 2.39 (m, 4H), 2.09 – 1.84 (m, 8H), 1.75 (dd, *J* = 13.8, 7.5 Hz, 8H), 1.67 – 1.40 (m, 8H), 1.22 (d, *J* = 6.2 Hz, 12H), 1.08 (d, *J* = 7.0 Hz, 12H).

#
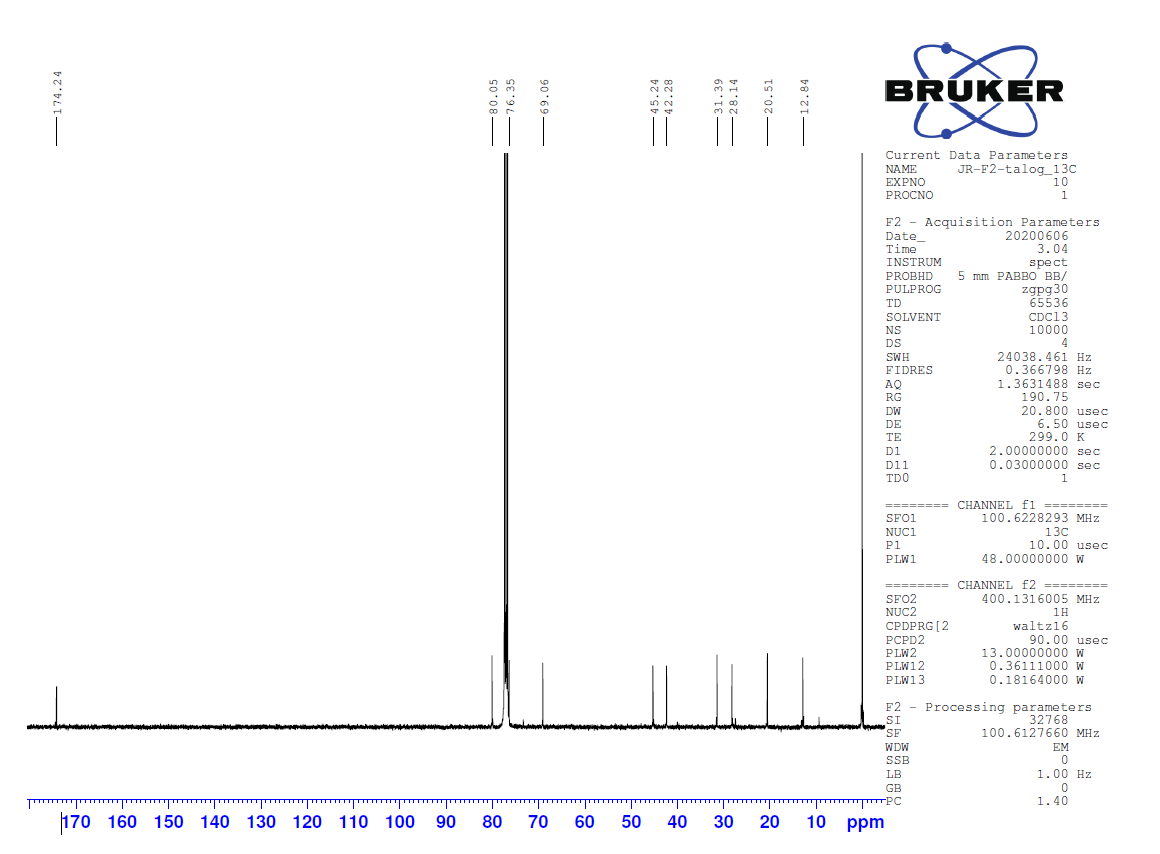


# Figure S6.^13^C spectrum of Nonactin (F2-precipitate). ^13^C NMR (101 MHz, CDCl_3_) δ 174.2, 80.0, 76.4, 69.1, 45.2, 42.3, 31.4, 28.1, 20.5, 12.8.

#
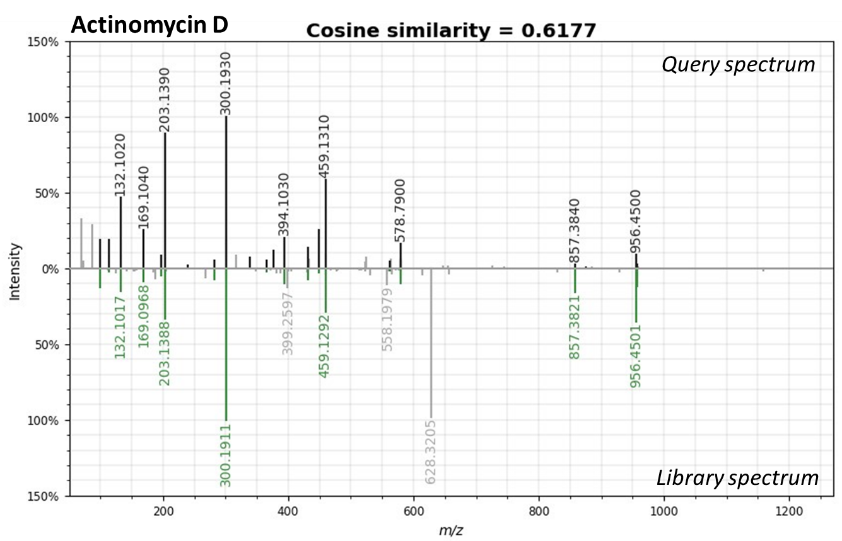

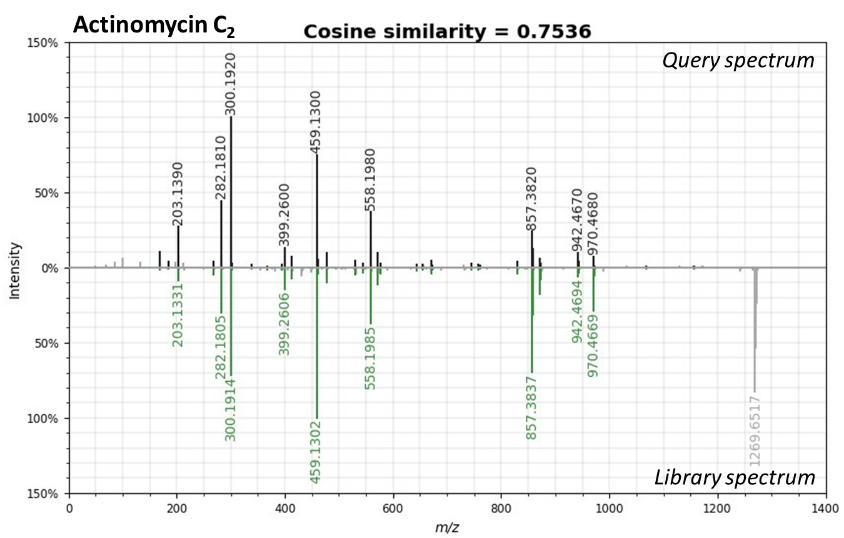


#
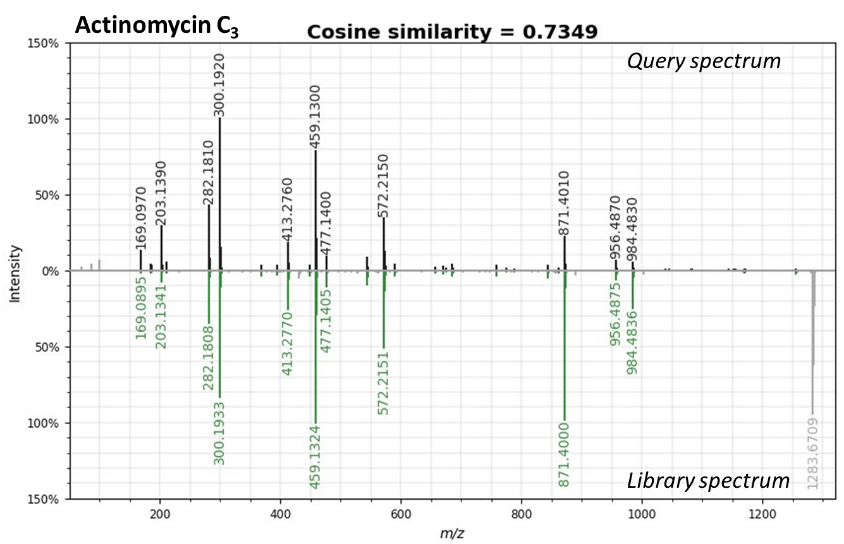


# Figure S7. LCMS/MS fragmentation mirror spectra compared to known fragmentation library of actinomycin D, actinomycin C2 and actinomycin C3.

#
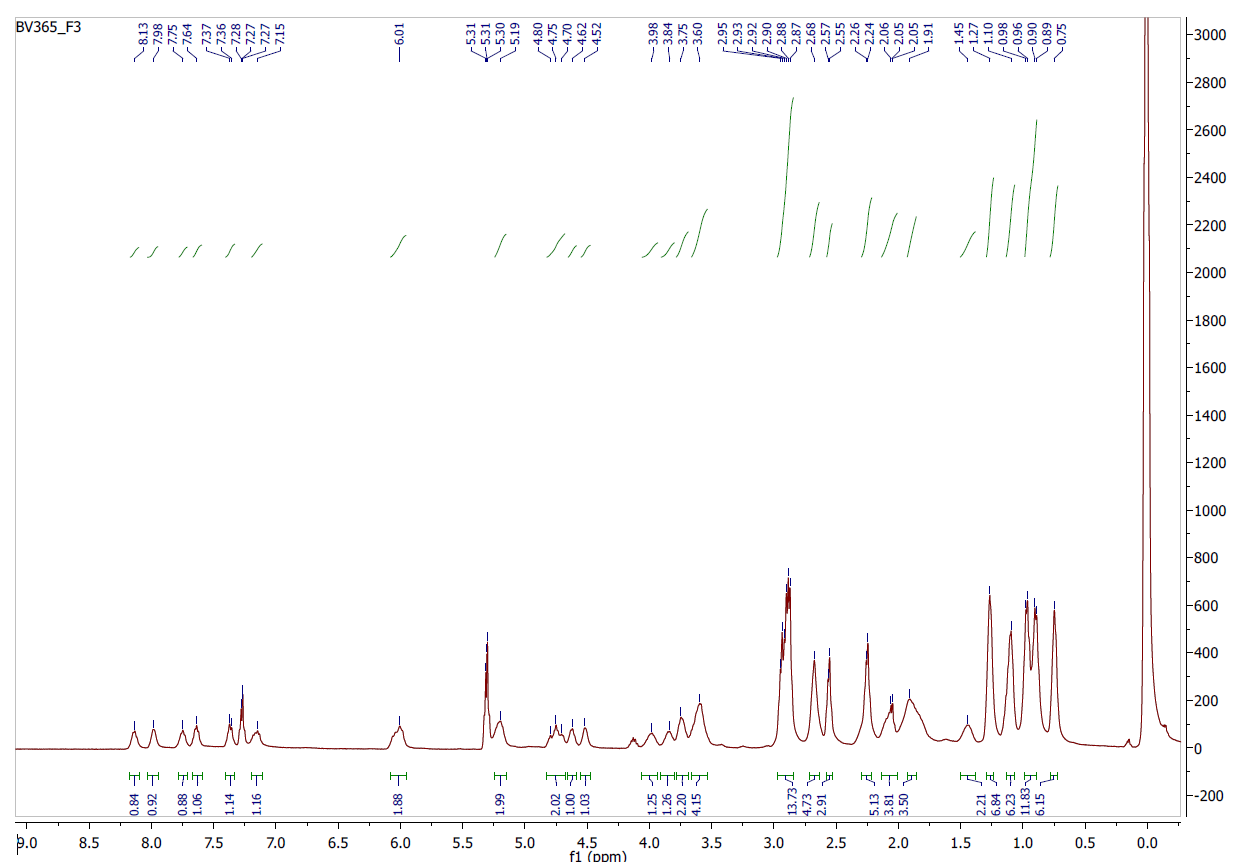


# Figure S8. ^1^H spectrum of Actinomycin D.^1^H NMR (400 MHz, CDCl3) δ 8.13 (s, 1H), 7.98 (s, 1H), 7.75 (s, 1H), 7.64 (s, 1H), 7.36 (d, J = 6.5 Hz, 1H), 7.15 (s, 1H), 6.01 (s, 2H), 5.19 (s, 2H), 4.75 (t, J = 18.2 Hz, 2H), 4.62 (s, 1H), 4.52 (s, 1H), 3.98 (s, 1H), 3.84 (s, 1H), 3.75 (s, 2H), 3.60 (s, 4H), 3.00 – 2.82 (m, 13H), 2.68 (s, 5H), 2.56 (d, J = 5.2 Hz, 3H), 2.25 (d, J = 5.1 Hz, 5H), 2.14 – 2.01 (m, 4H), 1.91 (s, 2H), 1.45 (s, 2H), 1.27 (s, 6H), 1.10 (s, 6H), 0.98-0.89 (m, 12H), 0.75 (s, 6H).

#
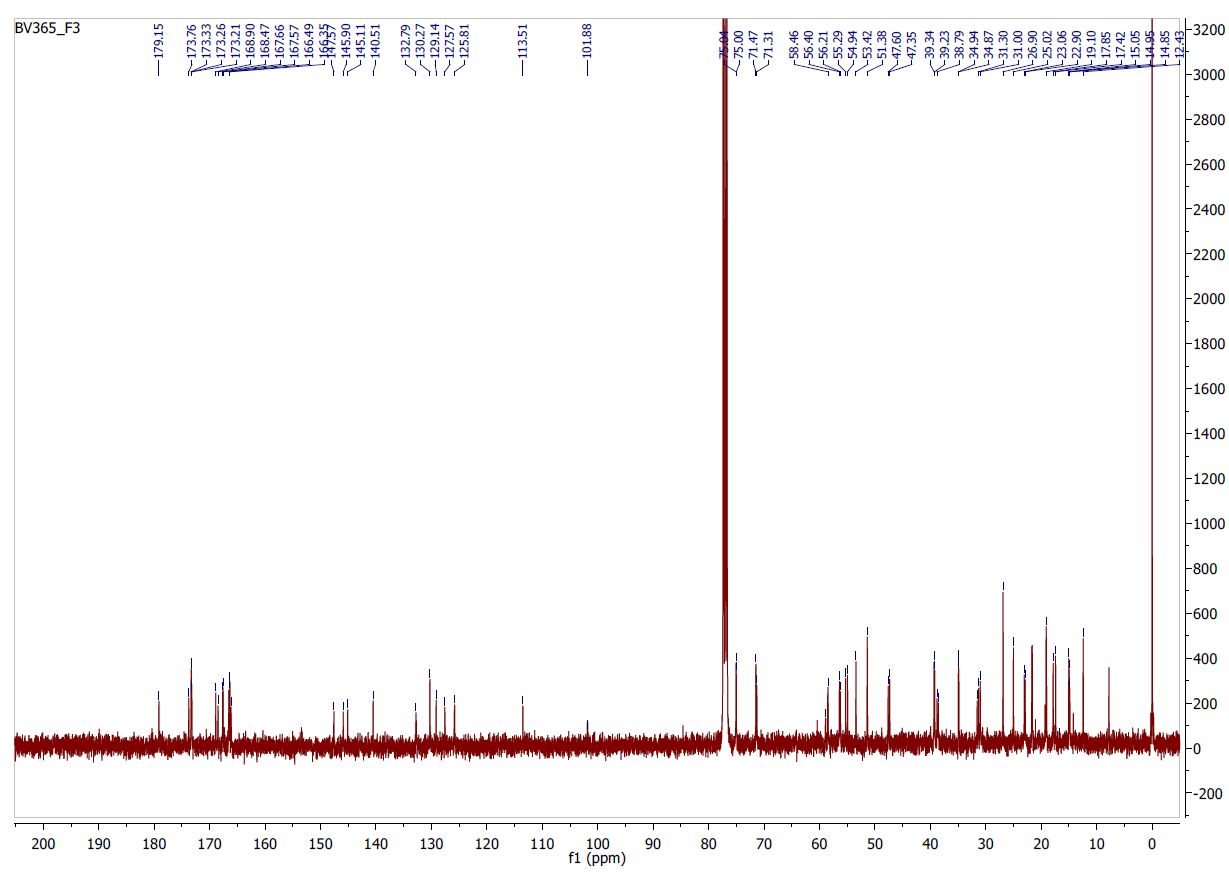


# Figure S9. ^13^C spectrum of Actinomycin D.^13^C NMR (101 MHz, CDCl3) δ 179.15, 173.76, 173.33, 173.26, 173.21, 168.90, 168.47, 167.66, 167.57, 166.56, 166.49, 166.35, 166.14, 147.57, 145.90, 145.11, 140.51, 132.79, 130.27, 129.14, 127.57, 125.81, 113.51, 101.71, 75.04, 75.00, 71.47, 71.31, 58.89, 58.46, 56.40, 56.21, 55.29, 54.94, 53.42, 51.38, 47.60, 47.35, 39.34, 39.23, 34.94, 34.87, 31.56, 31.30, 31.00, 26.90, 25.02, 23.06, 22.90, 19.10, 17.85, 17.42, 15.05, 14.95, 14.85, 12.43.

#

# Figure S10. UV-VIS spectra of *Streptomyces anulatus* BV365 actinomycins fraction F3 in acetone/water.

#

# Figure S11. Absorbance of the dyebath after dyeing of silk at 65 °C and pH 5 for different time.

# Figure S12. Color strength (K/S), color difference ΔE* and color coordinates (L*, a* and b*) of silk after one and three washing cycles.


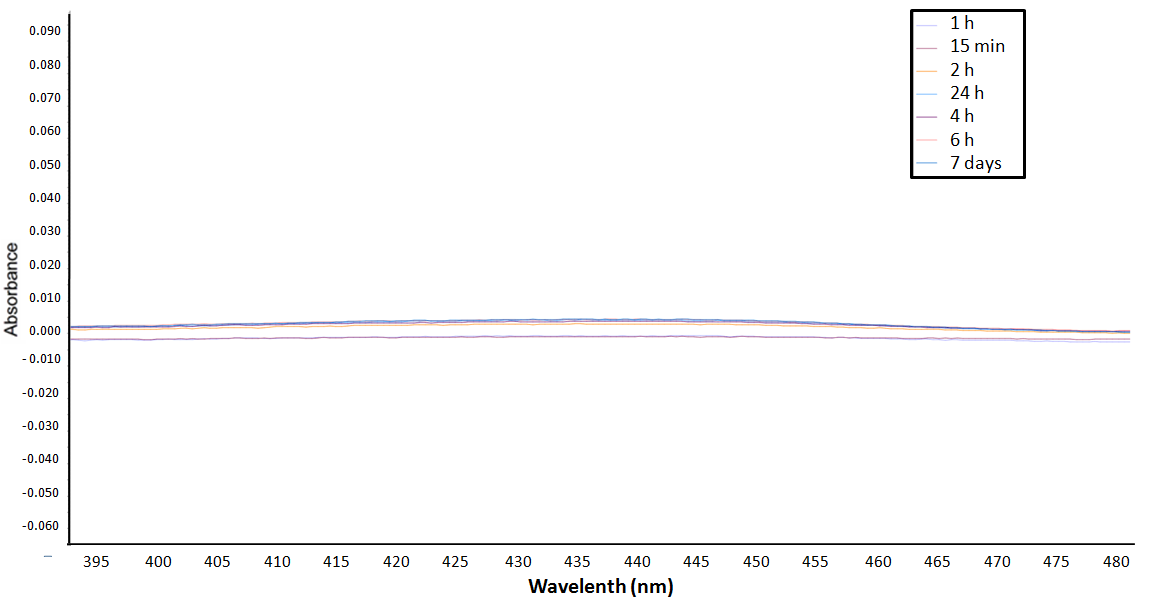


Figure S13. The release kinetics of the pigment from the dyed silk up to 7 days of keeping the fabric at 37 °C in PBS.

# Figure S14. Antibacterial activity of dyed silk fabrics colored during 10, 15, 30, 60 and 90 min, against *S. aureus* ATCC 25923, dynamic contact conditions.

# Table S1. Color coordinates and color strength of silk dyed at different concentration (0.5 and 1 % o.w.f.), temperature (25, 45, 65 and 85 °C) and pH (3, 5, 7 and 9) of the dyebath

| **sample** | **Conc, %** | **T, °C** | **pH** | **L** | **a** | **b** | **K/S** |
| --- | --- | --- | --- | --- | --- | --- | --- |
| **Silk 60* (multifiber fabric)** | 1 | 65 | 3 | 76.29 | 15.27 | 64.74 | 5.24 |
|  |  |  | 5 | 76.56 | 14.02 | 62.53 | 4.68 |
|  |  |  | 7 | 76.43 | 14.34 | 62.59 | 4.72 |
|  |  |  | 9 | 76.59 | 14.16 | 63.09 | 4.75 |
|  |  | 85 | 3 | 79.57 | 9.67 | 54.1 | 2.7 |
|  |  |  | 5 | 79.14 | 8.99 | 56.06 | 3 |
|  |  |  | 7 | 80.17 | 7.44 | 52.07 | 2.36 |
|  |  |  | 9 | 78.2 | 8.97 | 52.44 | 2.7 |
|  | 0.5 | 25 | 3 | 83.86 | 3.83 | 38.26 | 1.00 |
|  |  |  | 5 | 84.82 | 2.26 | 34.44 | 0.80 |
|  |  | 45 | 3 | 80.33 | 7.98 | 50.06 | 2.14 |
|  |  |  | 5 | 80.89 | 7.46 | 48.84 | 1.94 |
|  |  | 65 | 3 | 80.11 | 7.27 | 48.57 | 2.04 |
|  |  |  | 5 | 79.6 | 9.78 | 54.47 | 2.7 |
| **Silk 10 * (greige)** | 0.5 | 65 | 5 | 81.82 | 5.64 | 39.62 | 1.14 |
| **Silk 15* (greige)** |  |  |  | 81.55 | 5.79 | 41.63 | 1.28 |
| **Silk 30* (greige)** |  |  |  | 81.2 | 6.23 | 42.33 | 1.36 |
| **Silk 60* (greige)** |  |  |  | 80.85 | 6.19 | 44.58 | 1.57 |
| **Silk 90* (greige)** |  |  |  | 81.16 | 5.92 | 45.1 | 1.56 |

# * dyeing duration in min

# Table S2. Agar well diffusion assay. Zone of inhibition diameter (mm) of silk fabrics dyed during 10, 15, 30, 60 and 90 min against the microorganisms.

| Diameter of zone of inhibition (mm) | | | |
| --- | --- | --- | --- |
| F3 fraction/silk | *Staphylococcus aureus* MRSA ATCC 43300 | *Staphylococcus aureus* ATCC 25923 | *Staphylococcus aureus* 865 |
| 10’ | 3 | 3 | 3 |
| 15’ | 4 | 3 | 4 |
| 30’ | 3 | 3 | 3 |
| 60’ | 2 | 0.5 | 2.5 |
| 90’ | 3 | 3 | 3 |
| 60’ I wash | 2 | 3 | 3 |
| 60’ III wash | 0.5 | 0.5 | 1 |
| 90’ I wash | 0.5 | 0.5 | 2 |
| 90’ III wash | 1 | 1 | 1 |
